# Exploring the relationship between social capital and hedonic well-being in sport and physical activity contexts: a scoping review

**DOI:** 10.3389/fpsyg.2025.1540907

**Published:** 2025-02-13

**Authors:** Yi Zhang, Keita Kinoshita, Shintaro Sato

**Affiliations:** ^1^Sport & Entertainment Management Lab., Graduate School of Sport Sciences, Waseda University, Tokyo, Japan; ^2^Physical Education and Sport Science Department, National Institute of Education, Nanyang Technological University, Singapore, Singapore; ^3^Sport & Entertainment Management Lab., Faculty of Sport Sciences, Waseda University, Tokyo, Japan

**Keywords:** social capital, hedonic well-being, subjective well-being, sport, physical activity

## Abstract

This scoping review aimed to summarize the conceptualization and measurements of both social capital and hedonic well-being and to explore the links between social capital and hedonic well-being within sports and physical activity contexts. Articles were sourced from five databases, including PubMed, Scopus (Elsevier), SPORTDiscus, Web of Science, and Google Scholar. Initially, 475 papers were identified. After applying the screening process, 24 papers were included. The majority (70.8%) indicated a positive relationship between social capital and hedonic well-being, while others found no direct connection (16.7%) or presented mixed results (12.5%). The review underscored a consensus on defining and measuring hedonic well-being, but it also revealed the need for a more refined conceptualization and universally accepted measurement of social capital within sports research. The findings highlighted the positive associations between social capital and hedonic well-being in sport and physical activity contexts, suggesting future research directions including an examination of potential downsides.

## 1 Introduction

In recent years, the concept of social capital has increasingly gained prominence as a potential predictor of hedonic well-being. Emerging in the late 1980s, scholars have been exploring social capital from two distinct perspectives: collective and individual (Bourdieu, 1986). At the collective level, social capital encompasses features of social organization such as networks, norms, and social trust that bolster coordination and cooperation for mutual benefit (Putnam, 1995). Conversely, the individual perspective positions social capital as an individual’s social network or group membership which may yield economic, cultural, or symbolic capital or resources (Bourdieu, 1986, 2018). It can also be perceived as the social support or resources created through these social networks (Rodgers et al., 2019).

Parallel to this, the study of well-being, particularly hedonic well-being, has evolved as a critical area of inquiry within the sports context. Hedonic well-being, often operationalized as subjective well-being (SWB), encapsulates the presence or absence of positive feelings about life (Keyes, 2002) and has been extensively researched within the domain of sports (Lundqvist, 2011). However, an overarching review of the relationship between social capital and hedonic well-being within sports-related environments remains conspicuously absent, creating a gap that this scoping review aims to address.

Previous research has underscored the importance of social capital not only as a fundamental component necessary for the existence of a democratic society (Putnam, 2000; Harraka, 2002) but also as an integral determinant of physical and mental health (Rodgers et al., 2019). This dual role of social capital, benefiting both the society as a whole and individual well-being, is indispensable. Downward et al. (2018) reported a significant association between social capital and hedonic well-being at the individual level. At the collective level, social capital positively influenced hedonic well-being in society or one’s community (Matsushima and Matsunaga, 2015). At the practical level, the association between social capital and hedonic well-being can benefit both individuals and society. Understanding this relationship helps individuals to achieve successful aging or conquer autism (e.g., Bailey et al., 2020). Meanwhile, understanding social capital and hedonic well-being also benefits the event community through marathon events (e.g., Zhou et al., 2021). However, studies such as Kumar et al. (2019) contested this relationship, finding that social capital and hedonic well-being had no direct association but an indirect relationship mediating by health. The divergence in these findings underscores the complexity of defining and measuring social capital and hedonic well-being, especially within the context of sports and physical activities. This variation, in turn, accentuates the need for a scoping review of existing methodologies and outcomes within this realm.

The importance of physical activity, referring to any bodily movement produced by skeletal muscles resulting in energy expenditure (Caspersen et al., 1985), cannot be overstated, particularly its subset, sport, as a tool to augment public health and hedonic well-being (Downward et al., 2018). Moreover, sporting activities have been shown to enrich social capital by influencing various facets of social life (Kumar et al., 2019; Winkelmann, 2009). Physical exercise had an indirect positive association with hedonic well-being through social capital (Zhang et al., 2022). These findings are important especially to government and sport practitioners to enhance the public’s social capital and well-being. However, mixed results were found in online and offline sport participation (Lee et al., 2016). A thorough examination of the interplay between social capital and hedonic well-being in the context of physical activity and sport can offer significant insights and contribute to a more comprehensive understanding of the dynamics in this field.

Therefore, to bridge these gaps, this scoping review aims to accomplish three main objectives: (1) to conceptualize and measure social capital in sport and physical activity contexts, (2) to delineate the concept and measure of hedonic well-being in sport and physical activity contexts, and (3) to identify and synthesize the associations between social capital and hedonic well-being within sport and physical activity contexts. By achieving these objectives, this review will offer a robust framework for understanding and promoting hedonic well-being through social capital in sport and physical activity environments.

## 2 Methods

### 2.1 Protocol and registration

A scoping review is more suitable for this study due to its exploratory nature and the breadth of the field. We conducted a scoping review in order to understand the research scope of the topics and identify potential gaps. Scoping reviews, unlike systematic reviews, do not aim for comprehensive subject coverage (Dowling et al., 2018). Scholars also do not typically undertake an assessment of research quality (Dowling et al., 2018). As Arksey and O'Malley (2005) noted, scoping reviews are especially appropriate for topics that have not been extensively reviewed previously or when the extent of prior research in the area remains ambiguous. The link between social capital and hedonic well-being within the scope of sports and physical activities meets these conditions, as there exists a gap in the literature examining this interplay. We adopted Preferred Reporting Items for Systematic Reviews and Meta-Analyses extension for Scoping Reviews (PRISMA-ScR) as our drafting protocol, which was formulated by a research team with 24 experts under the guidance of EQUATOR (Enhancing the QUAlity and Transparency Of Health Research) Network (Tricco et al., 2018). The methodology stressed that a wide range of stakeholders should be involved and methodology rigor should be ensured by utilizing the checklist to report scoping reviews. Eventually, this methodology enables scoping reviews to have comprehensive coverage and gain clarity in reporting. The whole checklist for the current scoping review can be seen in the Appendix.

### 2.2 Eligibility criteria

Eligibility criteria were developed to evaluate identified articles. The inclusion criteria were published in journal articles with a formal hypothesis that studied the link between social capital and hedonic well-being, which contained at least one item of social capital and hedonic well-being measurements. We included all the research papers published from 1990 to 2023 (last research date: February 13th, 2023), for no review has been done regarding social capital and hedonic well-being in the sport and physical activity contexts. Studies were excluded if they (a) were not written in English, (b) did not focus on the relationship between social capital and hedonic well-being, (c) did not include empirical quantitative data, and (d) did not have a hypothesis on a statistical link between social capital and hedonic well-being. Only quantitative studies were included as the authors followed the positivism paradigm. The positive paradigm emphasizes the importance of objective and empirical data evidence, striving to minimize the influence of human bias in subjective interpretation (Alharahsheh and Pius, 2020).

### 2.3 Information sources

This scoping review utilized several databases, including PubMed, Scopus (Elsevier), SPORTDiscus, and Web of Science, to identify English-language articles published on or before the last searching date (February 13th, 2023). Google Scholar was used to do a grey search. The articles were identified utilizing a series of keywords of “social capital” and “well-being.” The keywords of “social capital” contained “social capital,” “social cohesion” and “social network.” The keywords of “hedonic well-being” contained “hedonic well-being” and “subjective well-being.” Since we were willing to explore social capital and hedonic well-being in sport and physical activity context, “sport,” “athlete(s)” and “physical activity” are also contained in the search strings. Besides, the grey search can be performed by manual search (Binepal et al., 2015). We used Google Scholar to search the references of the reviewed articles from the other four databases to identify more papers.

## 3 Results and discussion

### 3.1 Selection of sources of evidence

**Figure 1:**
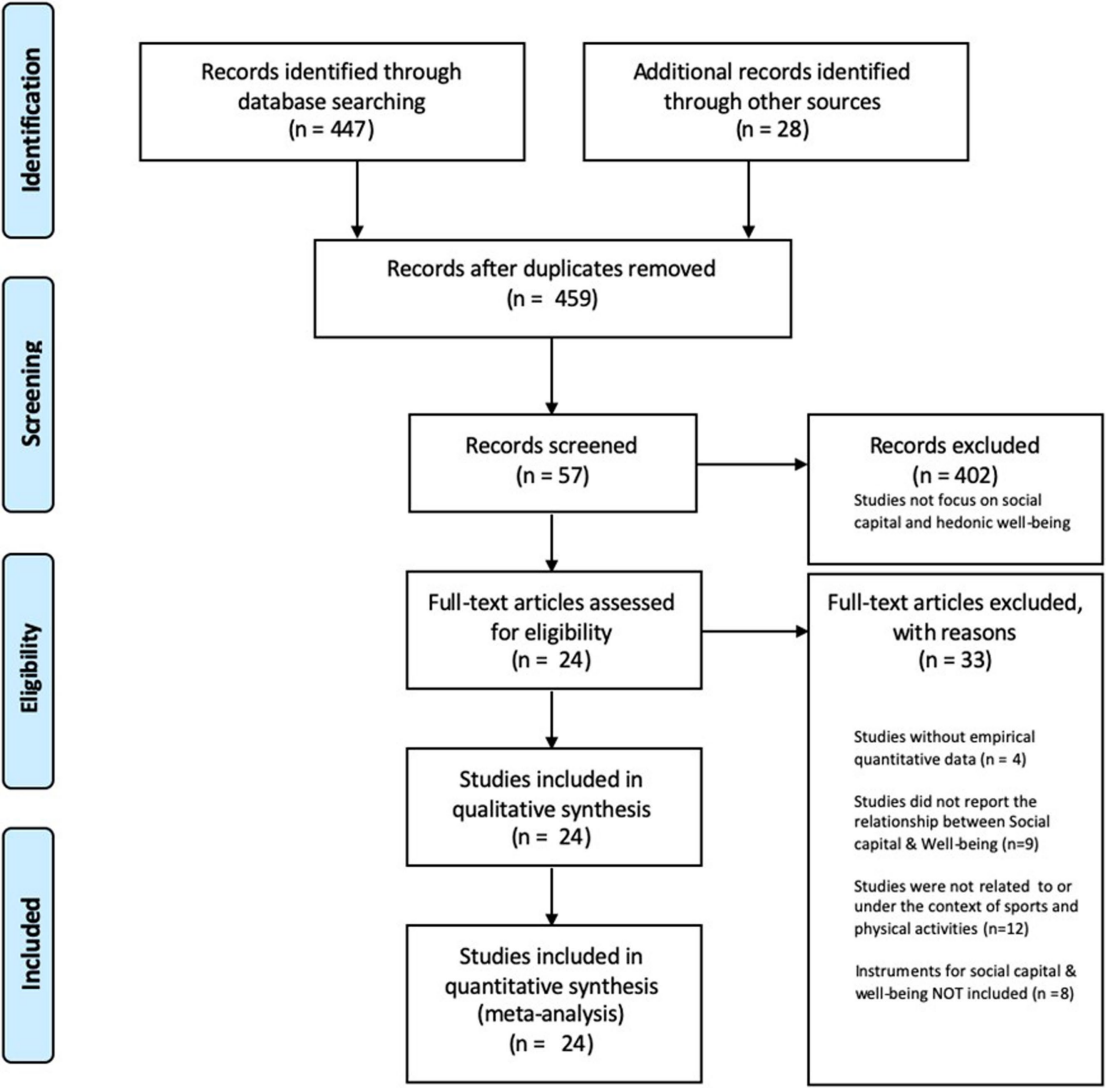
PRISMA flow diagram illustrating the screening process.

The screening process and the final screening results are illustrated in Figure 1. The initial search results from all the five databases we used were 475 articles. After applying the exclusion criteria, a total of 24 articles that met the eligibility criteria were included in the present study (refer to Figure 1).

### 3.2 Characteristics of sources evidence

Table 1 encapsulates the conceptualizations and measurements of social capital, and Table 2 presents an overview of the conceptualizations and measurements related to hedonic well-being. Table 3 provides the characteristics and key findings of each study in this scoping review, including region, study design, and main findings. The 24 studies collectively involved 129,669 participants, including 45,589 males, 54,826 females, and 29,254 with unidentified gender. The mean participant age, undisclosed in 10 studies, was approximately 46.45 years. Participant details per study are available in Table 3.

**Table 1:** Conceptualizations and the measurements for social capital.

Author (Year)	Conceptualization	Measurements
Ardahan (2018)	Social network	28-item Social Capital Scale for Turkey
Atilgan and Tükel (2021)	Social network	28-item Social Capital Scale for Turkey
Bartolini et al. (2008)	Social network	Three-type RSC; One-item Trust in institutions for Non-RSC
Huxhold et al. (2013)	Social network	One-item Network size; Nine-item Contact and frequency
Lee et al. (2016)	Social network	Four-item Perceived Social Support, 20-item Online and Offline Bonding and Bridging Scales
Lera-López et al. (2021)	Social network	Four-item Social Relationships with Family and Friends
Mcteer and Curtis (1993)	Social network	Seven-item Berkman Social Network Index
Niioka et al. (2020)	Social network	LSNS-6
Zhang et al. (2022)	Social network	Three-item Social Network Scale
Bailey et al. (2020)	Social support	Four item-FSS; Four-item Social Participation
Jeon et al. (2016)	Social support	25-item Social Support Scale
Mo et al. (2022)	Social support	Six-item Perceived Social Support Scale
Awaworyi Churchill and Mishra (2017)	Trust, network, norm	Six-item Social Network; Seven-item Trust
Lin (2022)	Trust, network, reciprocity	19-item Social Capital Scale
Matsushima and Matsunaga (2015)	Trust, social network, norms	One-item Trust; One-item Membership; One-item Volunteering
Taks and Rocha (2022)	Trust, network, norm	Four-item Social Cohesion
Winkelmann (2009)	Trust, network, norm	Six-item Social Engagement
Zhou et al. (2021)	Trust, social network, reciprocity	Nine-item SEPSCS
Kesavayuth et al. (2022a)	Social interaction, volunteer, membership	Three-item Social Capital Scale
Kesavayuth et al. (2022b)	Network, social involvement, trust	Three-item Social Capital Scale
Bjørnskov (2008)	Trust	One-item Trust; Two-type sociability
Downward et al. (2018)	Trust	One-item Trust
Kumar et al. (2019)	Trust	One-item Trust
Sun et al. (2021)	Social cohesion	15-item Group Environment Questionnaire

Source: Created by author.

**Table 2:** Conceptualizations and the measurements for hedonic well-being.

Author (Year)	Conceptualization	Measurements
Ardahan (2018)	Life satisfaction	Five-item Life Satisfaction Scale
Atilgan and Tükel (2021)	Life satisfaction	Five-item Life Satisfaction Scale for Turkey
Awaworyi Churchill and Mishra (2017)	Life satisfaction	One-item Life Satisfaction
Bartolini et al. (2008)	Happiness	One-item Reported Happiness
Bjørnskov (2008)	Happiness/ Life satisfaction	One-item Happiness/ Life Satisfaction
Downward et al. (2018)	Happiness/ Positive affect	One-item Happiness
Kesavayuth et al. (2022b)	Affect	Five-item Affect Scale
Kumar et al. (2019)	Happiness	One-item Happiness
Lera-López et al. (2021)	Happiness	One-item Happiness
Matsushima and Matsunaga (2015)	Happiness/ Life Satisfaction	One-item Generalized Happiness
Niioka et al. (2020)	Life satisfaction	Nine-item LSIK
Taks and Rocha (2022)	Life satisfaction	One-item Happiness; Three-item Life Satisfaction Scale; Eight-item Life Affect Scale
Zhang et al. (2022)	Life satisfaction	20-item Brief Subjective Well-Being Scale for Chinese Citizens
Zhou et al. (2021)	Life satisfaction	One-item Overall Life Satisfaction
Mcteer and Curtis (1993)	Psychological well-being	Four-item HAY Scale
Sun et al. (2021)	Life satisfaction, affect	Eight-item Subjective Exercise Experience Scale
Mo et al. (2022)	Life satisfaction, positive affect	Three-item Positive Feeling Subscale; One-item Life Satisfaction Scale
Lee et al. (2016)	Life Satisfaction, mental health	Five-item SWLS; Five-item RAND Mental Health Inventory
Kesavayuth et al. (2022a)	Life Satisfaction, mental health	One-item Life Satisfaction; MHI-5
Bailey et al. (2020)	Life Satisfaction, positive affect, negative affect	Five-item SWLS
Huxhold et al. (2013)	Life Satisfaction, positive affect, negative affect	Five-item SWLS; 20-item Emotional well-being: the Positive and Negative Affect
Jeon et al. (2016)	Life Satisfaction, positive affect, negative affect	Five-item SWLS; 22-item Korean Emotional Experience Scale
Lin (2022)	Life Satisfaction, positive affect, negative affect	Two-item Subjective Well-being
Winkelmann, 2009	Life satisfaction, positive affect, negative affect	One-item General Life Satisfaction

Source: Created by author.

**Table 3:** Characteristics and main findings in sport-context studies.

Author (Year)	Sample *n* (M/F/other) *M*_age_	Region	Study design	Main findings (related to sports)
Ardahan (2018)	Volunteers and non-volunteers Total *n* = 420 Volunteers *n* = 208 (162, 46, 0) N/A Non-volunteers *n* = 138 (111, 27, 0) N/A	Turkey	A cross-sectional study	Social capital had a positive association with life satisfaction. Volunteers preferred to enjoy sportive activities, but non-volunteer people liked passive activities. Volunteers had a higher social capital value and thus a higher life satisfaction value compared with non-volunteers.
Atilgan and Tükel (2021)	Coaches *n* = 251 (185, 66, 0) N/A	Turkey	A cross-sectional study	Participants’ social capital perceptions a positive and significant association with their life satisfaction. Individuals who actively participate in sports showed notably higher scores in their perceptions of social capital compared to those who did not actively participate in sports.
Awaworyi Churchill and Mishra (2017)	Chinese Residence *n* = 6,731 (3,231, 3,500, 0) *M*_age_ = 41.56 years	China	A cross-sectional study	Social networks and trust have a positive link with SWB. In China, trust related to family members and neighbors has a positive association with SWB, yet trust related to other groups of people have a weaker association with SWB. Social networks generated from sport groups had a significant positive relationship with SWB.
Bailey et al. (2020)	Autistic university students Online survey: *n* = 42 (28, 13, 1) *M*_age_ = 21.76 Interview: *n* = 20 (14, 5, 1) *M*_age_ = 22.90	US	A mixed methods study	Perceived social support has a positive relationship with SWB. However, participating in sports as an item of social capital did not have a significant association with well-being, but time spent hanging out with friends.
Bartolini et al. (2008)	US residents *n* = 46,510 (20,436, 26,074, 0) *M*_age_ = 45.3 years	US	A cross-sectional study	A significant and positive correlation exits between several social capital indicator and happiness. Intrinsic RSC has a positive correlation with happiness, but extrinsic RSC has a negative correlation with happiness. Non-RSC has a positive association with happiness.
Bjørnskov (2008)	US residents N/A	US	A cross-sectional study	Social trust has a positive association with happiness, yet the informal sociability only possibly has weak relevant to happiness. A negative but insignificant association exists between formal sociability and happiness.
Downward et al. (2018)	UK residence Wave 4: *n* = 14,452 (N/A) Wave 6: *n* = 14,102(N/A) N/A	UK	Half: A rolling cross-sectional sample; The other half: A longitudinal web-based survey	The authors identified a clear positive and significant association between social capital and SWB. SWB has a stronger influence on sport through social capital. Generally, sport and social capital has no obvious relationship.
Huxhold et al. (2013)	Older German residence *n* = 2034 (1,060, 974, 0) *M*_age_ = 73.72 years	German	A cohort-sequential design	Social network indicated by network structure had no direct influence on SWB, but had indirect effects on SWB via improving emotional support and social activity engagement like visiting sports events or doing sports. The change of social activity engagement mediated the relationship between the changes of social network and the changes of SWB.
Jeon et al. (2016)	Elite student athletes from high school or universities *n* = 333 University: (123, 21, 0) *M*_age_ = 21.5 years High school students: (131, 58,0) *M*_age_ = 17.9 years	Korean	A cross-sectional design	Social support had a positive association with SWB in both direct and indirect ways. Social support and SWB were partially mediated by self-compassion.
Kesavayuth et al. (2022a)	Australian Residents n = 17,428 (8,247, 9,181, 0) N/A	Australia	A cross-sectional study	Social capital positively predicts SWB, and being an active membership in a sport club has a positive impact on life satisfaction, but not on mental health. The frequency of participating in physical activities has a positive relationship with SWB.
Kesavayuth et al. (2022b)	Australian Elder Residents n = 4,955 (2,279, 2,676, 0) *M*_age_ = 65.56 years	Australia	A cross-sectional study	Hedonic well-being had a positive effect on social capital and the frequency of physical activities.
Kumar et al. (2019)	UK residence *n* = 361 (143, 218, 0) N/A	UK	A cross-sectional design	Social capital and SWB had an indirect association. Social capital had a positive impact on health and then on SWB. Sport and fitness activity participation can directly influence social capital and health, and consequently influence well-being.
Lee et al. (2016)	Undergraduate students *n* = 574 (236, 336, 2) *M*_age_ = 20.6 years	US	A cross-sectional design	Bonding social capital had a constant relationship with SWB in off-line context, while bridging social capital only had a significant association with overall life satisfaction in online context. Sport participation had a positive impact on SWB and social support when social capital is taken into consideration.
Lera-López et al. (2021)	Spain people n = 1,632 (801, 831, 0) *M*_age_ = 47.4 years	Spain	A cross-sectional study	Social relationships with family have a significant positive relationship with happiness, while social relationships with friends have no significant correlation with happiness. Sport participation is positively related to happiness. The correlation between passive sport involvement and personal happiness seems to be stronger than the correlation between active sport participation and personal happiness.
Lin (2022)	Taiwan baseball spectators n = 422 (213, 209, 0) N/A	Taiwan, China	A cross-sectional study	Social capital is positively related to SWB under a professional baseball spectating context.
Matsushima and Matsunaga (2015)	Japanese residence *n* = 1,674 (812, 862, 0) N/A	Japan	A cross-sectional design	Overall, social capital had a positive association with SWB. Trust and volunteering had a positive relationship with SWB, but membership of sport associations did not show a statistical significance.
Mcteer and Curtis (1993)	American residence Wave 1 *n* = 3,025, (1,102, 1,685, 238) N/A Wave 2 *n* = 2,436 (665, 1,387, 393) N/A	US	A two-wave cross-sectional design	For both males and females, social capital and SWB had fairly strong positive associations. Sport and physical activities had a positive association with social capital, but they only had a significant relationship with SWB for women.
Mo et al. (2022)	Hong Kong local residents n = 408 (104, 304, 0) *M*_age_ = 44.31 years	China	A cross-sectional study	Social support has a positive association with SWB. Physical activities mediate the relationship between social support and SWB among the younger people, but not for the elder people.
Niioka et al. (2020)	Users of day care rehabilitation services *n* = 123 (39, 84, 0) *M*_age_ = 78.46 years	Japan	A cross-sectional study	Social capital was not identified an association with hedonic well-being in the rehabilitation context.
Sun et al. (2021)	Chinese female who participate in a square dance group n = 1,166 (0, 1,166, 0) *M*_age_ > 50 years	China	A cross-sectional study	SWB generated from participating in a dance group has a strong and positive relationship with group cohesion.
Taks and Rocha (2022)	Host country residents Time 1 n = 402 (222, 180, 0) *M*_age_ = 27.9 years Time 2 n = 401 (213, 188, 0) *M*_age_ = 29.4 years	Brazil	A cohort longitudinal design	Social cohesion had no significant impact on SWB during and after the 2016 Rio Olympic Games.
Winkelmann (2009)	German residents *n* = 5,536 (3,059, 2,477, 0) *M*_age_ = 38.5 years	German	A cross-sectional study	Social capital had a positive and significant influence on SWB, and sport is the most influential social activities based on quantitative support.
Zhang et al. (2022)	Chinese residents n = 4,031 (1872, 2,159, 0) *M*_age_ = 50.75 years	China	A cross-sectional study	Social networks positively predicted SWB and played a mediating role between the association of physical activities and SWB. Physical activities had a positive relationship with both social networks and SWB.
Zhou et al. (2021)	Running event participants *n* = 200 (101, 99, 0) *M*_age_ = 35 years	US	A cross-sectional study	Trust and reciprocity had significant positive relationship with overall life satisfaction, but network did not show significant influence on overall life satisfaction. Participants’ life satisfaction may be mainly driven by trust and reciprocity.

Source: Created by author.

### 3.3 Conceptualization of social capital in sport and physical activity contexts

Prior research acknowledges the lack of consensus in defining social capital (Bartolini et al., 2008; Rodgers et al., 2019). This review identified five interpretations of social capital in the context of sport. Specifically, 37.5% (9/24) studies defined social capital as resources derived from social networks (Atilgan and Tükel, 2021; Ardahan, 2018; Bartolini et al., 2008; Huxhold et al., 2013; Lee et al., 2016; Lera-López et al., 2021; Mcteer and Curtis, 1993; Niioka et al., 2020; Zhang et al., 2022). Another eight studies were in line with Putnam's (1995, 2000) three-dimensional definition: social networks, norms, and trust (Awaworyi Churchill and Mishra, 2017; Kesavayuth et al., 2022a; Kesavayuth et al., 2022b; Lin, 2022; Matsushima and Matsunaga, 2015; Taks and Rocha, 2022; Winkelmann, 2009; Zhou et al., 2021). Trust was considered central to social capital in four studies (Bartolini et al., 2008; Bjørnskov, 2008; Downward et al., 2018; Kumar et al., 2019), while three viewed social support as the prime indicator (Bailey et al., 2020; Jeon et al., 2016; Mo et al., 2022). Sun et al. (2021) represented social capital through social cohesion.

Overall, we identified both single-dimensional and multidimensional ways to conceptualize social capital in the sport and physical activity contexts (Table 1). The mainstream concept of social capital is to operationalize social capital as resources produced from social networks (nine papers), and social support is considered as one of the resources (three papers). Meanwhile, six papers used Putnam’s (1995, 2000) concept of social capital focuses on trust, social network(s) and reciprocity/norms. This is another popular way to define social capital based on the results of this review. This conclusion matches the findings of the previous review of social capital (e.g., Rodgers et al., 2019). Besides, Kesavayuth et al. (2022a) included *volunteering* as one of the three indicators of social capital. Matsushima and Matsunaga (2015) measured norms by volunteering. Participants were asked whether they “have done volunteering in the past year.” Ardahan (2018) conceptualized social capital within a network of volunteers. Social capital can be effectively fostered through sport volunteerism (Perks, 2007), yet there is room to further explore hedonic well-being through sport volunteerism.

### 3.4 Measurement of social capital in the sport and physical activity contexts

We found 17 measurements for social capital (Table 1), including (1) a Generalized Trust; (2) the Japanese version of the abbreviated Lubben Social Network Scale (LSNS-6; Kurimoto et al., 2011); (3) Berkman Social Network Index (Berkman and Syme, 1979); (4) Social Network Scale (Li and Chen, 2012); (5) Social Relationship with family and friends (Center for Sociological Research, 2014); (6) Social Support Scale (Park, 1985; Yun, 1993); (7) the Perceived Social Support Scale (Zimet et al., 1988); (8) the Perceived Social Support Scale (Su et al., 2014); (9) The Family Support Scale (FSS; Dunst et al., 1988); (10) Online and Offline Bonding and Bridging scales (Williams, 2006), (11) Social Capital Scale for Turkey (Ardahan, 2012; Onyx and Bullen, 2000), (12) Social Capital Scale (Melbourne Institute of Applied Economic and Social Research, 2018), (13) Social Cohesion (Taks and Rocha, 2017), (14) Social Capital Scale (Gibson et al., 2014), (15) Group Environmental Questionnaire (Carron et al., 1985; Hongyu, 2008), (16) Social Engagement (German Institute for Economic Research, 2004) and (17) Sport Event Participation Social Capital Scale (SEPSCS; Zhou et al., 2021).

Researchers measuring social capital in a single-dimensional manner focused on social network, support, trust, or cohesion. To measure social networks, the Social Capital Scale for Turkey (Ardahan, 2012; Onyx and Bullen, 2000) was utilized by Ardahan (2018) and Atilgan and Tükel (2021) for social network characteristics. This scale has been adapted for various cultures including Greece and China (Kritsotakis et al., 2008; Xu et al., 2020). Bartolini et al. (2008) used Relational Social Capital and Trust in Institutions, respectively, to test two different types of social capital, while Huxhold et al. (2013) measured network size, contact, and frequency. Lee et al. (2016) measured Online and Offline Bonding and Bridging Scales (Williams, 2006) and Perceived Social Support (Zimet et al., 1988) for social network. Other notable scales include the four-item Social Relationships with Family and Friends (Lera-López et al., 2021), the seven-item Berkman Social Network Index (Mcteer and Curtis, 1993), and the six-item Japanese version of LSNS-6 for elder rehabilitation (Niioka et al., 2020). Zhang et al. (2022) used the three-item Social Network Scale (Li and Chen, 2012). Limitations were noted in only measuring social network size (Matsushima and Matsunaga, 2015; Zhou et al., 2021), for it cannot measure other key characteristics of social network, such as social engagement or contact frequency (e.g., Huxhold et al., 2013; Winkelmann, 2009). Social support was measured by Jeon et al. (2016) using the 25-item Social Support Scale (Park, 1985; Yun, 1993), Bailey et al. (2020) with the Family Support Scale (FSS; Dunst et al., 1988), and Mo et al. (2022) used a six-item Perceived Social Support Scale (Su et al., 2014). Bailey et al. (2020) discussed that FSS is more suitable when targeting young people.

In the multidimensional approach to social capital, six papers adopted Putnam’s definition, focusing on trust, networks, and reciprocity/community participation. Awaworyi Churchill and Mishra (2017) used the six-item Social Network and seven-item Trust from the World Values Survey (WVS). Lin (2022) used a 19-item Social Capital Scale with trust, safety, social connections, and collective action indicators. Matsushima and Matsunaga (2015) used single-item measures for trust, membership, and volunteering, discussing the insufficiency of their membership measure due to its focus on network size rather than active participation. Despite their definition, Taks and Rocha (2022) measured only social cohesion in the context of Rio 2016. Winkelmann (2009) used the six-item Social Engagement scale (German Institute for Economic Research, 2004), measuring participation in networks and events. Lastly, Zhou et al. (2021) developed SEPSCS to measure trust, network, and reciprocity, providing researchers with a specific way to test sport participants’ social capital in the context of sport events.

Fourteen papers conducted research in the sport and physical activity participation contexts, including sport or physical activities participation (Ardahan, 2018; Atilgan and Tükel, 2021; Bailey et al., 2020; Downward et al., 2018; Jeon et al., 2016; Kesavayuth et al., 2022a; Kesavayuth et al., 2022b; Mcteer and Curtis, 1993; Mo et al., 2022; Niioka et al., 2020; Sun et al., 2021; Winkelmann, 2009; Zhang et al., 2022) and sport participation events (i.e., Marathon events; Zhou et al., 2021). The majority of the papers have concentrated on social network and resources generated from social network, which is a more individualized way of measuring social capital. However, sport event participation like Marathon events were measured in a more collective way by using Putnam’s concept (1995) and SEPSCS. Overall, the participation type of context focused more on social networks, which implies the personal network and its benefits are highly valued in these contexts. Although the authors used various ways to measure social network, we suggest not only network size should be measured, but also other related aspects like contact and frequencies should be tested (e.g., Huxhold et al., 2013). A Generalized Trust and Online and Offline Bonding and Bridging scales can be used to operationalize trust or social network, respectively, in both online and offline sport participation contexts.

Five research (Bjørnskov, 2008; Kumar et al., 2019; Huxhold et al., 2013; Lee et al., 2016; Lera-López et al., 2021) were conducted in both sport participation and sport spectatorship contexts. Four studies out of five used a single-dimensional way to conceptualize social capital, including trust (Bjørnskov, 2008; Kumar et al., 2019) and social network/ relationships (Lera-López et al., 2021; Huxhold et al., 2013). Singular-dimensional ways to indicate social capital suggested a focused interest of how these singular aspects work in the overall social capital impacting hedonic well-being.

Two research were done in the sport spectatorship context (Lin, 2022; Taks and Rocha, 2022). Researchers all used Putnam’s concept to define social capital. Lin (2022) used a 19-item Social Capital Scale, and Taks and Rocha (2022) measured social cohesion. Three studies were done in the context of belonging to sport groups (Awaworyi Churchill and Mishra, 2017; Bartolini et al., 2008; Matsushima and Matsunaga, 2015). All of the three studies were in line with Putnam’s (1995, 2000) way of conceptualizing social capital, and their focus was measuring trust, social network/social cohesion and reciprocity/volunteering in a more collective way. Social Capital Scale (Onyx and Bullen, 2000; Gibson et al., 2014) can be a sufficient measurement, for this is a multi-dimensional scale that includes trust, network and reciprocity. SEPSCS (Zhou et al., 2021) can be also utilized to test social capital in sport spectatorship context as sport spectatorship is a passive sport participation. The two scales are aligned with Putnam (1995)‘s social capital concept.

### 3.5 Conceptualization of hedonic well-being in sport and physical activity context

Hedonic well-being (SWB) is considered a multifaceted concept, primarily defined as self-rated life satisfaction, positive affect, and negative affect within sports and physical activity contexts (Diener, 1984; Diener et al., 1985; Diener et al., 2017; Diener et al., 2018). Eight studies in this review adhered to this definition (Atilgan and Tükel, 2021; Bailey et al., 2020; Jeon et al., 2016; Kesavayuth et al., 2022b; Niioka et al., 2020; Sun et al., 2021; Taks and Rocha, 2022; Zhang et al., 2022), while others also aligned with Diener’s core elements (Huxhold et al., 2013; Lin, 2022; Mo et al., 2022; Winkelmann, 2009). Additionally, six studies viewed happiness or life satisfaction as indicators of SWB (Bartolini et al., 2008; Bjørnskov, 2008; Downward et al., 2018; Kumar et al., 2019; Lera-López et al., 2021; Zhou et al., 2021), two equated SWB with positive feelings or satisfaction (Ardahan, 2018; Awaworyi Churchill and Mishra, 2017), two used SWB synonymously with happiness and life satisfaction (Matsushima and Matsunaga, 2015; Mcteer and Curtis, 1993), and two more defined it as life satisfaction and mental health (Kesavayuth et al., 2022a; Lee et al., 2016). In summary, 62.5% (15 out of 24) of the articles used life satisfaction, happiness, affect, or psychological well-being as measures of hedonic well-being (Table 2). Nine of these 15 papers employed life satisfaction, while one used psychological well-being. Other studies took a multidimensional approach: five (20.8%) conceptualized hedonic well-being as life satisfaction, positive affect, and negative affect; two (8.3%) regarded it as a combination of life satisfaction and affect; and another two (8.3%) defined it as life satisfaction and mental health. Thus, the majority of sport and physical activity research conceptualizes hedonic well-being primarily through a single factor, like life satisfaction or happiness. Eight research (33.3%) conceptualized hedonic well-being by Diener’s work (Diener, 1984, 1994, 2000; Diener et al., 1985, 2017, 2018). The findings were in line with Huta and Waterman (2014), where most studies assessed hedonic well-being using one or more indicators of subjective well-being (i.e., life satisfaction, positive affect, and/or negative affect).

### 3.6 Measurements of hedonic well-being in the sport and physical activity context

We identified 16 measurements of testing hedonic well-being in sport and physical activity context (Table 2), including (1) Satisfaction with Life Scale (SWLS; Diener et al., 1985), (2) the 20-item Positive and Negative Affect (Watson et al., 1988), (3) the 22-item Korean Emotional Experience Scale (Hong, 2004), (4) the Life Satisfaction Index K (LSIK; Koyano, 1990), (5) the five-item RAND Mental Health Inventory (Berwick et al., 1991; Stewart et al., 1992), (6) the four-item HAY (How are you?) Scale (National Center for Health Statistics, 1982), (7) one-item Overall Life Satisfaction (Helliwell and Putnam, 2004; Kavetsos and Szymanski, 2010), (8) a single item Happiness, (9) the 20-item Brief Subjective Well-Being Scale for Chinese Citizens (Xing, 2003), (10) the five-item Mental Health Inventory (MHI-5) (Rumpf et al., 2001), (11) the five-item Affect Scale (Melbourne Institute of Applied Economic and Social Research, 2018), (12) the three-item Life Satisfaction Scale (Connolly, 2013), (13) the eight-item Life Affect Scale (Diener, 2000), (14) the three-item Positive Feeling (Su et al., 2014), (15) the two-item Subjective Well-being (Portela et al., 2013), and (16) the eight-item Positive Subjective Exercise Experience Scale (SEES; MeAuley and Courneya, 1994).

Matched with the conceptualization of hedonic well-being, we found that the main way of measuring hedonic well-being was by using a single-item happiness/ life satisfaction (50%). The other main way of measuring hedonic well-being was by Diener et al. (1985) SWLS (5 items). Six research (25%) utilized this scale. Besides SWLS, two research measured participants’ affect by using the Positive and Negative Affect Scale and the Korean Emotional Experience Scale (Huxhold et al., 2013; Jeon et al., 2016). Mental health was adopted by researchers (Kesavayuth et al., 2022a; Lee et al., 2016) as one of the indicators of hedonic well-being by using the RAND Mental Health Inventory (Berwick et al., 1991; Stewart et al., 1992) and MHI-5 (Rumpf et al., 2001). Mcteer and Curtis (1993) used the Four-item HAY (How are you?) Scale (National Center for Health Statistics, 1982), a measurement of psychological well-being, to test subjective well-being. Sun et al. (2021) utilized four-item psychological well-being to represent the positive subjective feeling. However, mental health and psychological well-being are regarded as core indicators for eudaimonic well-being in previous research (Huta and Waterman, 2014).

In sport and physical activity participation contexts, researchers used life satisfaction and/or affect, which was matched with Diener’s way of conceptualizing hedonic well-being. Five-item Life Satisfaction Scale (Diener et al., 1985) was the main way to measure hedonic well-being in the sport participation context (Ardahan, 2018; Atilgan and Tükel, 2021; Bailey et al., 2020; Jeon et al., 2016). In the physical activity context, Niioka et al. (2020) used the Life Satisfaction Index K (LSIK) to evaluate subjective well-being in terms of life satisfaction, psychological state, and old age assessment targeting the elder people who took rehabilitation. In the Chinese square dance context, Sun et al. (2021) employed an eight-item SEES (MeAuley and Courneya, 1994) to evaluate elder female participants’ psychological well-being and psychological fatigue. Zhang et al. (2022) utilized a 20-item Brief Subjective Well-Being Scale for Chinese Citizens (Xing, 2003) for elder Chinese residents. These studies offer scales for assessing hedonic well-being in older populations.

All of the four research in sport participation and sport spectatorship contexts used happiness/ life satisfaction to indicate hedonic well-being, and one item-happiness/ satisfaction was the main measurement (Bjørnskov, 2008; Kumar et al., 2019; Lera-López et al., 2021). Matching with social capital in sport participation and sport spectatorship contexts, hedonic well-being also confronted a trend with a single-dimensional way of measurement. Similarly, in the context of belonging to sport groups, all of the three papers used happiness/ life satisfaction to conceptualize and test hedonic well-being.

In sport spectatorship context, researchers conceptualized hedonic well-being by life satisfaction and/or affect. Lin (2022) used two-item Subjective Well-being (Portela et al., 2013) to measure spectators’ SWB during the 2020 Chinese Professional Baseball League (CPBL) season. Taks and Rocha (2022), studying Rio 2016, measured hedonic well-being by using one-item Happiness (Kavetsos and Szymanski, 2010), three-item Life Satisfaction Scale (Connolly, 2013), and eight-item Life Affect Scale (Diener, 2000). These two research provided scales to measure SWB in spectating sport events. While the measurement in participatory sport is more related to life satisfaction, researchers studied sport spectating context also measured affect during and after the events.

### 3.7 The relationships between social capital and hedonic well-being in the sport and physical activity contexts

Of the 24 articles in this review, 17 (70.8%) showed a positive association between social capital and hedonic well-being, four (16.7%) identified no direct relationship, and three (12.5%) reported mixed results (Table 3).

For the findings related to the impact of sports and physical activities, 13 studies (54.2%) reported a positive association among sport-related factors, social capital and/or hedonic well-being, four studies (16.7%) reported no obvious influence from sport or physical activities, and three studies (12.5%) reported mixed results. Two out of the 13 studies reported hedonic well-being positively impacted the frequency of participation in physical activities (Downward et al., 2018; Kesavayuth et al., 2022b). Other studies (Jeon et al., 2016; Lin, 2022; Niioka et al., 2020; Zhou et al., 2021) did not report the influence of sport-related activities, but their targets or contexts were related to sports or physical activities.

For the 17 studies indicating positive relationships between social capital and hedonic well-being, one (5.9%) evaluated social cohesion, two (11.8%) gauged social trust, three (17.6%) assessed social support, and four (23.5%) examined social networks. Seven studies (41.2%) utilized three indicators to measure social capital, with five out of these seven adopting Putnam’s definition. These five found a positive correlation between social capital and hedonic well-being, although two reported that networks (or memberships) did not show statistical significance (Matsushima and Matsunaga, 2015; Zhou et al., 2021). Matsushima and Matsunaga (2015) highlighted the inadequate measurement of membership, and Zhou et al. (2021) suggested examining bonding and bridging social capital in sports event contexts. Based on the research included in this review (Huxhold et al., 2013; Winkelmann, 2009), it is recommended to measure social networks not only by size but also by contact frequency and social engagement.

Among the four studies reporting no direct relationship between social capital and hedonic well-being, Kumar et al. (2019) found an indirect association mediated by health, while Huxhold et al. (2013) and Niioka et al. (2020) reported indirect associations through emotional support and social activity engagement, and no correlation in a rehabilitation context, respectively. Notably, the latter two focused on older individuals, emphasizing the importance of social network size and engagement for successful aging. In future research with similar target groups, broader measurements of social capital are recommended. Taks and Rocha (2022), defining social capital by Putnam (2000), found no significant influence of social cohesion on subjective well-being post-Rio 2016, suggesting a more multidimensional approach, including measurements of trust and norm, could better indicate social capital.

For mixed results, Lee et al. (2016) found offline bonding social capital consistently related to life satisfaction and mental health, while online bridging social capital was only significantly associated with overall life satisfaction. Bartolini et al. (2008) revealed a positive relationship between non-relational social capital and hedonic well-being, but a negative correlation with extrinsic-motivated Relational Social Capital (RSC). This study underscored the role of motivations when assessing social capital. Lera-López et al. (2021) found that family relationships, but not friendships, were positively associated with happiness, highlighting the mediating role of social groups in social capital and hedonic well-being relationships. Furthermore, the double-edged nature of social capital, as discussed by Villalonga-Olives and Kawachi (2017), calls for more research into its negative effects on hedonic well-being in sport and physical activity contexts.

The included articles in this review provided empirical evidence for the relationships between social capital and hedonic well-being in various sport and physical activity contexts (Table 4). These studies advanced knowledge regarding how social capital associated with hedonic well-being through sport and physical activities. Zhou et al. (2021) validated Sport Event Participation Social Capital Scale, offering a reliable measurement for social capital outcomes through sport event participation. This study also revealed that marathon participants’ social capital positively relates to hedonic well-being. Kesavayuth et al. (2022b) tested the mediating role of social capital. Social capital and physical activity were two significant mediators between hedonic well-being and elder residents’ physical health. Policymakers can enhance the elders’ well-being and physical health through physical activity through social capital and physical activity (Kesavayuth et al., 2022b). These studies highlighted the importance of social capital and physical activity on individuals’ hedonic well-being. Researchers also tested the sport spectatorship (i.e., passive sport participation). Lee et al. (2016) reported bridging social capital had a positive relationship with hedonic well-being when people spectated basketball game, football game, and other sport events online. Lera-López et al. (2021) found that passive sport participation (i.e., spectating sport events) had a higher correlation with hedonic well-being compared with active physical activity participation (i.e., walking). Lin (2022) found that professional baseball spectators’ social capital positively was associated with hedonic well-being. These studies showed that passive sport participation can play an important role when developing an individual’s social capital and hedonic well-being. However, the studies included in the scoping review were conducted within a single country (Table 3), limiting the generalization across diverse cultural contexts. Nineteen studies only utilized a cross-sectional study design (Table 3), so potential biased inferences may exist (Bowen and Wiersema, 1999). Future research should focus more on the multi-cultural contexts and longitudinal study design.

**Table 4:** Specific sport-contexts in this scoping review.

Author (Year)	Sport and physical activity
Ardahan (2018)	Sportive activities in leisure times, including outdoor sports, water sports, fitness sports, team sports, and motor sports.
Atilgan and Tükel (2021)	Sport participation: active or not
Awaworyi Churchill and Mishra (2017)	Whether the respondents belong to a sport group
Bailey et al. (2020)	Frequency of participation in sports, including club, intramural, or varsity
Bartolini et al. (2008)	Membership in sports clubs
Bjørnskov (2008)	Sport-related activities, measured as informal sociability, including attending a sporting event, going swimming, going bowling and playing tennis.
Downward et al. (2018)	Total minutes of sport activity over past 4 weeks
Huxhold et al. (2013)	Sports-related activities in social activities: visiting sport events; doing sports
Jeon et al. (2016)	Elite student athletes’ training at schools
Kesavayuth et al. (2022a)	Physical activities: the frequency of physical activity and the number of hours spending on outdoor tasks in a week.
Kesavayuth et al. (2022b)	The frequency of physical activity: “In general, how often do you participate in moderate or intensive physical activity for at least 30 min?”
Kumar et al. (2019)	Total minutes of sport and physical activities in the last four-week period: 8 h week at sport facilities during the four-week period on average, as well as 3 to 5 h of other physical activities in this period
Lee et al. (2016)	Sports participation: basketball game, football game, and other sport events in both online and off-line ways
Lera-López et al. (2021)	Sport participation and frequency: Active (walking) and passive (watching sport events)
Lin (2022)	A Professional Baseball Franchise Context: Spectating Brothers Elephants during the 2020 CPBL season
Matsushima and Matsunaga (2015)	Whether an individual is a member of sports associations
Mcteer and Curtis (1993)	The frequency of physical activities, including swimming, dancing, gardening, jogging, running, riding a bike, calisthenics or physical exercise, other active sports.
Mo et al. (2022)	Physical activities: doing exercises for three times a week.
Niioka et al. (2020)	Day care rehabilitation services
Sun et al. (2021)	Fitness activity: Chinese square dance
Taks and Rocha (2022)	Sport mega-events: Rio 2016 Olympic and Paralympic Games (Rio 2016)
Winkelmann (2009)	Engaging actively in sports as one of the six social activities
Zhang et al. (2022)	Physical exercise: “In the past 12 months, how many times per week did you normally perform up to 30 min of physical activity that made you sweat?” A continuous variable with 0–96.
Zhou et al. (2021)	Sport event participation: Participation in running events per year and overall running event experience

Source: Created by author.

### 3.8 Limitations and future directions

This scoping screened papers without quantitative data based on exclusion criteria to gain sufficient quantitative support and identify the measurement of social capital and hedonic well-being. In addition, this scoping review only covered papers written and published in English, which possibly led to language bias. Lastly, since 83.3% of studies in this review were cross-sectional (Table 3), future research should adopt a longitudinal design to better substantiate the relationship between social capital and hedonic well-being across time.

The conceptual vagueness of social capital (Bjørnskov, 2008) prompts both single and multi-dimensional measurements. Single-dimensional measurements, often seen in panel survey data studies, are time-efficient yet potentially risky due to over-reliance on social networks. Matsushima and Matsunaga (2015) and Zhou et al. (2021) suggest that when assessing social networks, factors such as frequency, density, and engagement should also be considered, not just network size. Hedonic well-being, as defined by Diener (1994) and Downward et al. (2018), encompasses life satisfaction, positive affect, and negative affect. Despite this, only two out of the eight studies in this review that used Diener’s definition measured positive and negative affects alongside life satisfaction. Therefore, future studies should consider comprehensive measurements of hedonic well-being, using tools such as the Satisfaction with Life Scale (SWLS) (Diener et al., 1985), and the Positive and Negative Affect Schedule (PANAS) (Watson et al., 1988; Thompson, 2007). In sport and physical activity contexts, the relationship between social capital and hedonic well-being is primarily positive. However, some studies reported no direct relationship or mixed results, especially when participants have harsh health conditions (Kumar et al., 2019; Niioka et al., 2020). Hence, it is crucial to delve into these variances in future research. The exploration of the potential downside of social capital or negative social capital within sports and physical activities can also enrich our understanding of its influence in these fields. Therefore, this review calls for further comprehensive research on social capital and hedonic well-being, highlighting the need for more nuanced measurements and considerations of different contexts within the sport and physical activity sphere.

Future studies could focus more on differentiating the role of bonding and bridging social capital. Only one study measured and discussed bonding and bridging social capital in the online and offline sport contexts in the scoping review. Further, as four studies reported no direct relationship between social capital and hedonic well-being (Huxhold et al., 2013; Kumar et al., 2019; Niioka et al., 2020; Taks and Rocha, 2022), future research could conduct more research with the mediators for the relationship. This scoping review identified *social engagement* (Huxhold et al., 2013), *emotional support* (Huxhold et al., 2013), *self-compassion* (Jeon et al., 2016), *health-promoting behaviors* (Mo et al., 2022), and *income level* (Sun et al., 2021) as mediators. Huxhold et al. (2013) discussed that an individual’s social network structure (i.e., network size and contact frequency) was found to have no direct effects on SWB, whereas it appears to influence health and SWB indirectly by enhancing emotional support and engagement in social activities. Jeon et al. (2016) reported that self-compassion played a partially mediating role between social support and SWB. Mo et al. (2022) revealed the mediating role of health-promoting behaviors (e.g., physical activities) between social support and SWB for young people under 35. Sun et al. (2021) found out income level played a partial mediation role in their research as subjective exercise experience still predicted group cohesion significantly with controlling income level as a mediator. Future researchers could test the detailed mechanism between social capital and hedonic well-being with these mediators in sport and physical activity contexts.

As mixed results were reported (Bartolini et al., 2008; Lee et al., 2016; Lera-López et al., 2021), future research could identify potential moderators that may moderate the relationship between social capital and hedonic well-being in the sport contexts. For the moderators, we identified age (Matsushima and Matsunaga, 2015) and other possible moderators, including *positive/ passive lifestyle* (Ardahan, 2018), *formal/ informal groups* (Awaworyi Churchill and Mishra, 2017; Bjørnskov, 2008), sports/ *leisure activity* (Bailey et al., 2020), *intrinsic/ extrinsic motivation* (Bartolini et al., 2008) and *medical health* (Mo et al., 2022). Matsushima and Matsunaga (2015) reported that age categories can moderate the relationship between volunteering, one of the indicators of social capital, and happiness. They uncovered the moderating role of the age category between volunteering and happiness. Individuals in their 50s who have volunteered are more likely to report lower levels of happiness compared to those who are aged 60 or above (Matsushima and Matsunaga, 2015, p. 1041). For possible moderators, according to Ardahan (2018), individuals who volunteered tended to prefer engaging in active sports and physical activities, whereas non-volunteers tended to prefer more passive leisure activities. Volunteers exhibited higher levels of social capital and reported higher levels of life satisfaction compared to non-volunteers. Awaworyi Churchill and Mishra (2017) identified that membership in sport groups and self-help groups had a significant relationship with well-being, whereas the membership in professional, educational and church groups did not contain significant association. Bjørnskov (2008) captured sociability from formal and informal activities. Formal activities are organized (e.g., volunteer work, club meetings, and church attendance), while informal activities (e.g., picnics, camping, and sport-related activities) are not needed to be pre-planned or formally organized. They reported that informal sociability contained a weak relevance to happiness, while formal sociability had a negative but insignificant relationship with happiness. Bailey et al. (2020) detected that social participation generated from leisure activity had a significant and positive association with well-being for autistic university students, but participation in sports was negatively related to well-being. Bartolini et al. (2008) discovered social capital generated from intrinsic groups (Putnam’s groups) had a positive association with happiness, while social capital generated from extrinsic groups (Olson’s groups) contained a negative relationship with happiness. Putnam’s groups included groups that people attended due to their intrinsic motivation, such as sport clubs, national organizations, and hobby clubs; Olson’s groups contained groups that people participated because of extrinsic motives, such as fraternity organizations, professional organizations and unions (Putnam, 1995; Olson, 1982; Bartolini et al., 2008). These results suggested that the moderating role of intrinsic motivation and extrinsic motivations can be further explored. An individual’s physical health and mental health may moderate the role between social capital and hedonic well-being (Mo et al., 2022). These potential moderators could be explored between social capital and hedonic well-being in sport and physical activity contexts.

## 4 Conclusion

This scoping review of 24 papers revealed two primary definitions of social capital: one focused on social networks or resources derived from them, and the other aligned with Putnam’s definition, which conceptualized social capital not only by social network but also by trust and reciprocity. Both single and multidimensional approaches were used. Hedonic well-being was typically conceptualized based on Diener’s work, with SWLS being a frequently utilized scale. Out of the studies, 70.8% identified a positive relationship between social capital and hedonic well-being, 16.7% found no direct link, and 12.5% reported mixed results. Despite the consensus on defining hedonic well-being, the need for standardized measurement of social capital in sports research was evident. The findings mainly spotlighted the positive links between social capital and hedonic well-being in sports and physical activities, suggesting further research, including potential negatives.

## Data Availability

The original contributions presented in the study are included in the article/Supplementary material, further inquiries can be directed to the corresponding author.

## References

[ref1] Alharahsheh H. H. Pius A. (2020). A review of key paradigms: positivism VS interpretivism. Glob. Acad. J. Hum. Soc. Sci. *2*, 39–43. doi: 10.36348/gajhss.2020.v02i03.001

[ref2] Ardahan F. (2012). Adaption of “social capital scale”: validity and reliability study. J. Hum. Sci. 9, 773–789.

[ref3] Ardahan F. (2018). Comparison of the social capital, life satisfaction, achievement perception and emotional intelligence level of the volunteers and non-volunteers. Eur. J. Phys. Educ. Sport Sci. 4, 45–68. doi: 10.46827/ejpe.v0i0.1656

[ref4] Arksey H. O'Malley L. (2005). Scoping studies: towards a methodological framework. Int. J. Soc. Res. Methodol. 8, 19–32. doi: 10.1080/1364557032000119616

[ref5] Atilgan D. Tükel Y. (2021). Social capital and satisfaction with life during the COVID-19 pandemic: a case study on coaches. Int. J. Soc. Educ. Sci. 3, 342–359. doi: 10.46328/ijonses.185

[ref6] Awaworyi Churchill S. Mishra V. (2017). Trust, social networks and subjective wellbeing in China. Soc. Indic. Res. 132, 313–339. doi: 10.1007/s11205-015-1220-2

[ref7] Bailey K. M. Frost K. M. Casagrande K. Ingersoll B. (2020). The relationship between social experience and subjective well-being in autistic college students: a mixed methods study. Autism 24, 1081–1092. doi: 10.1177/1362361319892457 31845592

[ref8] Bartolini S. Bilancini E. Pugno M. (2008). American declines of social capital and happiness: Is there any linkage. Siena: University of Siena.

[ref9] Berkman L. F. Syme S. L. (1979). Social networks, host resistance, and mortality: a nine-year follow-up study of Alameda County residents. Am. J. Epidemiol. 109, 186–204. doi: 10.1093/oxfordjournals.aje.a112674 425958

[ref10] Berwick D. M. Murphy J. M. Goldman P. A. Ware J. E. Jr. Barsky A. J. Weinstein M. C. (1991). Performance of a five-item mental health screening test. Med. Care 29, 169–176. doi: 10.1097/00005650-199102000-00008 1994148

[ref11] Binepal N. Lemyre B. Dunn S. Daboval T. Aglipay M. Leduc S. . (2015). Systematic review and quality appraisal of international guidelines on perinatal care of extremely premature infants. Curr. Pediatr. Rev. 11, 126–134. doi: 10.2174/1573396311666150608125529 26050792

[ref12] Bjørnskov C. (2008). Social capital and happiness in the United States. Appl. Res. Qual. Life 3, 43–62. doi: 10.1007/s11482-008-9046-6

[ref13] Bourdieu P. (1986). Distinction: A social critique of the judgement of taste. Harvard university press. Abingdon, UK: Routledge.

[ref14] Bourdieu P. (2018). “The forms of capital” in The sociology of economic life (New York: Routledge), 78–92.

[ref15] Bowen H. P. Wiersema M. F. (1999). Matching method to paradigm in strategy research: limitations of cross-sectional analysis and some methodological alternatives. Strateg. Manag. J. 20, 625–636. doi: 10.1002/(SICI)1097-0266(199907)20:7<625::AID-SMJ45>3.0.CO;2-V

[ref16] Carron A. V. Widmeyer W. N. Brawley L. R. (1985). The development of an instrument to assess cohesion in sport teams: the group environment questionnaire. J. Sport Exerc. Psychol. 7, 244–266. doi: 10.1123/jsp.7.3.244

[ref17] Caspersen C. J. Powell K. E. Christenson G. M. (1985). Physical activity, exercise, and physical fitness: definitions and distinctions for health-related research. Public Health Rep. 100, 126–131. 3920711 PMC1424733

[ref18] Center for Sociological Research. (2014). Barómetro de Junio 2014. Available at: http://www.cis.es/cis/opencm/ES/1_encuestas/estudios/ver.jsp?estudio=14090 (Accessed January 7, 2022).

[ref19] Connolly M. (2013). Some like it mild and not too wet: the influence of weather on subjective well-being. J. Happiness Stud. 14, 457–473. doi: 10.1007/s10902-012-9338-2

[ref20] Diener E. (1984). Subjective well-being. Psychol. Bull. 95, 542–575. doi: 10.1037/0033-2909.95.3.542 6399758

[ref21] Diener E. (1994). Assessing subjective well-being: Progress and opportunities. Soc. Indic. Res. 31, 103–157. doi: 10.1007/BF01207052

[ref22] Diener E. (2000). Subjective well-being: the science of happiness and a proposal for a national index. Am. Psychol. 55, 34–43. doi: 10.1037/0003-066X.55.1.34 11392863

[ref23] Diener E. D. Emmons R. A. Larsen R. J. Griffin S. (1985). The satisfaction with life scale. J. Pers. Assess. 49, 71–75. doi: 10.1207/s15327752jpa4901_13 16367493

[ref24] Diener E. Lucas R. E. Oishi S. Hall N. Donnellan M. B. (2018). Advances and open questions in the science of subjective well-being. *Collabra*. Psychology 4:115. doi: 10.1525/collabra.115 PMC6329388 30637366

[ref25] Diener E. Pressman S. D. Hunter J. Delgadillo-Chase D. (2017). If, why, and when subjective well-being influences health, and future needed research. Appl. Psychol. Health Well Being 9, 133–167. doi: 10.1111/aphw.12090 28707767

[ref26] Dowling M. Leopkey B. Smith L. (2018). Governance in sport: a scoping review. J. Sport Manag. 32, 438–451. doi: 10.1123/jsm.2018-0032

[ref27] Downward P. Hallmann K. Rasciute S. (2018). Exploring the interrelationship between sport, health and social outcomes in the UK: implications for health policy. Eur. J. Public Health 28, 99–104. doi: 10.1093/eurpub/ckx063 28510694

[ref28] Dunst C. J. Jenkins V. Trivette C. M. (1988). “Family support scale” in Enabling and empowering families: Principles and guidelines for practice. eds. Dunst C. J. Trivette C. M. Deal A. G. (Cambridge, MA: Brookline Books), 153–174.

[ref29] German Institute for Economic Research (2004). Socio-economic panel (SOEP), data for years 1984–2004. Berlin: DIW.

[ref30] Gibson H. J. Walker M. Thapa B. Kaplanidou K. Geldenhuys S. Coetzee W. (2014). Psychic income and social capital among host nation residents: a pre–post analysis of the 2010 FIFA world cup in South Africa. Tour. Manag. 44, 113–122. doi: 10.1016/j.tourman.2013.12.013

[ref31] Harraka M. (2002). Bowling alone: the collapse and revival of American community, by Robert D Putnam. J. Catholic Educ. 6:13. doi: 10.15365/joce.0602122013

[ref32] Hong C. H. (2004). Validation study of the Korean emotional experience scale. Korean J. Clin. Psychol. 23, 771–787.

[ref33] Hongyu M. (2008). The revise of the group environment questionnaire. J. Beijing Sport Univ. 3, 339–342. doi: 10.19582/j.cnki.11-3785/g8.2008.03.017

[ref34] Huta V. Waterman A. S. (2014). Eudaimonia and its distinction from hedonia: developing a classification and terminology for understanding conceptual and operational definitions. J. Happiness Stud. 15, 1425–1456. doi: 10.1007/s10902-013-9485-0

[ref35] Huxhold O. Fiori K. L. Windsor T. D. (2013). The dynamic interplay of social network characteristics, subjective well-being, and health: the costs and benefits of socio-emotional selectivity. Psychol. Aging 28, 3–16. doi: 10.1037/a0030170 23066804

[ref36] Jeon H. Lee K. Kwon S. (2016). Investigation of the structural relationships between social support, self-compassion, and subjective well-being in Korean elite student athletes. Psychol. Rep. 119, 39–54. doi: 10.1177/0033294116658226 27381414

[ref37] Kavetsos G. Szymanski S. (2010). National well-being and international sports events. J. Econ. Psychol. 31, 158–171. doi: 10.1016/j.joep.2009.11.005

[ref38] Kesavayuth D. Binh Tran D. Zikos V. (2022a). Locus of control and subjective well-being: panel evidence from Australia. PLoS One *17*:e0272714. doi: 10.1371/journal.pone.0272714 36044403 PMC9432765

[ref39] Kesavayuth D. Shangkhum P. Zikos V. (2022b). Well-being and physical health: a mediation analysis. J. Happiness Stud. *23*, 2849–2879. doi: 10.1007/s10902-022-00529-y

[ref40] Keyes C. L. (2002). The mental health continuum: from languishing to flourishing in life. J. Health Soc. Behav. 43:207. doi: 10.2307/3090197 12096700

[ref41] Koyano W. (1990). Structure of a life satisfaction index: invariability of factorial structure. Jpn. J. Gerontol. 12, 102–116.

[ref42] Kritsotakis G. Koutis A. D. Alegakis A. K. Philalithis A. E. (2008). Development of the social capital questionnaire in Greece. Res. Nurs. Health 31, 217–225. doi: 10.1002/nur.20250 18213683

[ref43] Kumar H. Downward P. Hodgkinson I. Manoli A. E. (2019). Means as well as ends: some critical insights for UK sport policy on the impact of facility ownership and configuration on sports participation. Int. J. Sport Policy Politics 11, 415–432. doi: 10.1080/19406940.2018.1522660

[ref44] Kurimoto A. Awata S. Ohkubo T. Tsubota-Utsugi M. Asayama K. Takahashi K. . (2011). Reliability and validity of the Japanese version of the abbreviated Lubben social network scale. Nihon Ronen Igakkai zasshi. Jpn. J. Geriatr. 48, 149–157. doi: 10.3143/geriatrics.48.149 21778631

[ref45] Lee S. Chung J. E. Park N. (2016). Linking cultural capital with subjective well-being and social support: the role of communication networks. Soc. Sci. Comput. Rev. 34, 172–196. doi: 10.1177/0894439315577347

[ref46] Lera-López F. Ollo-López A. Sánchez-Santos J. M. (2021). Is passive sport engagement positively associated with happiness? Appl. Psychol. Health Well Being 13, 195–218. doi: 10.1111/aphw.12227 33022139

[ref47] Li S. Chen G. (2012). Can Guanxi bring rural residents happiness? With evidence from rural China. Chin. Rural Econ. 8, 66–78.

[ref48] Lin Y. H. (2022). Antecedents and outcomes of social capital: evidence from a professional baseball franchise. Psychol. Res. Behav. Manag. 15, 261–272. doi: 10.2147/PRBM.S338512 35177943 PMC8846623

[ref49] Lundqvist C. (2011). Well-being in competitive sports—the feel-good factor? A review of conceptual considerations of well-being. Int. Rev. Sport Exerc. Psychol. 4, 109–127. doi: 10.1080/1750984X.2011.584067

[ref50] Matsushima M. Matsunaga Y. (2015). Social capital and subjective well-being in Japan. Volunt. Int. J. Volunt. Nonprofit Org. 26, 1016–1045. doi: 10.1007/s11266-015-9581-3

[ref51] Mcteer W. Curtis J. (1993). Sport and physical activity and subjective well-being: national panel data for the US. Int. Rev. Sociol. Sport 28, 397–412. doi: 10.1177/101269029302800404

[ref52] MeAuley E. Courneya K. S. (1994). The subjective exercise experiences scale (SEES): development and preliminary validation. J. Sport Exerc. Psychol. 16, 163–177. doi: 10.1123/jsep.16.2.163

[ref53] Melbourne Institute of Applied Economic and Social Research (2018). Household, income and labour dynamics in Australia (HILDA) survey - GENERAL RELEASE 17 (waves 1-17). Parkville, VIC: The University of Melbourne.

[ref54] Mo P. K. Wong E. L. Yeung N. C. Wong S. Y. Chung R. Y. Tong A. C. . (2022). Differential associations among social support, health promoting behaviors, health-related quality of life and subjective well-being in older and younger persons: a structural equation modelling approach. Health Qual. Life Outcomes *20*:38. doi: 10.1186/s12955-022-01931-z 35246166 PMC8895671

[ref55] National Center for Health Statistics (1982). National Survey of personal health practices and consequences (US), 1979–1980. Hyattsville, MD: NCHS.

[ref56] Niioka Y. Tsushima E. Ogihara H. Sato T. Hirayama K. Taguchi T. (2020). Factors related to quality of life in elderly users of day care rehabilitation services: an investigation using health-related quality of life and subjective well-being. Hirosaki Medical Journal 70, 130–138. doi: 10.32216/hirosakiigaku.70.2-4_130

[ref001] Olson M. (1982). The rise and decline of nations : economic growth, stagflation, and social rigidities (1st ed.). Yale University Press.

[ref57] Onyx J. Bullen P. (2000). Measuring social capital in five communities. J. Appl. Behav. Sci. 36, 23–42. doi: 10.1177/0021886300361002

[ref58] Park J. W. (1985). The research for developing social support scale (doctoral dissertation). Seoul, South Korea: Yonsei University of graduation.

[ref59] Perks T. (2007). Does sport foster social capital? The contribution of sport to a lifestyle of community participation. Sociol. Sport J. 24, 378–401. doi: 10.1123/ssj.24.4.378

[ref60] Portela M. Neira I. Salinas-Jiménez M. D. M. (2013). Social capital and subjective wellbeing in Europe: a new approach on social capital. Soc. Indic. Res. 114, 493–511. doi: 10.1007/s11205-012-0158-x

[ref61] Putnam R. D. (1995). Bowling alone: America’s declining social capital. J. Democr. 6, 65–78. doi: 10.1353/jod.1995.0002

[ref62] Putnam R. D. (2000). Bowling alone: The collapse and revival of American community. New York: Simon and Schuster.

[ref63] Rodgers J. Valuev A. V. Hswen Y. Subramanian S. V. (2019). Social capital and physical health: an updated review of the literature for 2007–2018. Soc. Sci. Med. 236:112360. doi: 10.1016/j.socscimed.2019.112360 31352315

[ref64] Rumpf H. J. Meyer C. Hapke U. John U. (2001). Screening for mental health: validity of the MHI-5 using DSM-IV Axis I psychiatric disorders as gold standard. Psychiatry Res. 105, 243–253. doi: 10.1016/S0165-1781(01)00329-8 11814543

[ref65] Stewart A. L. Ware J. E. Sherbourne C. D. Wells K. B. (1992). “Psychological distress/well-being and cognitive functioning measures” in Measuring functioning and well-being: The medical outcomes study approach. eds. Stewart A. L. Ware J. E. Jr. (Durham, NC: Duke University Press), 102–142.

[ref66] Su R. Tay L. Diener E. (2014). The development and validation of the comprehensive inventory of thriving (CIT) and the brief inventory of thriving (BIT). Appl. Psychol. Health Well Being *6*, 251–279. doi: 10.1111/aphw.12027 24919454

[ref67] Sun Y. Ji P. Wang Y. Fan H. (2021). The association between the subjective exercise experience of Chinese women participating in square dance and group cohesion: the mediating effect of income. Front. Psychol. 12:700408. doi: 10.3389/fpsyg.2021.700408 34712166 PMC8546298

[ref68] Taks M. Rocha C. (2017). “Maakte Rio 2016 de Brazilianen gelukkig? [Did Rio 2016 make the Braz ilians happy?]” in The story of Rio 2016: De maatschappelijke betekenis van de olympische en paralympische spelen 2016 [the story of Rio 2016: The societal meaning of the Olympic and Paralympic games 2016]. eds. Hoover P. Breedveld K. (Utrecht, Netherlands: Mulier Instituut), 119–127.

[ref69] Taks M. Rocha C. (2022). Involvement, social impacts and subjective well-being: Brazilians’ experiences from Rio 2016 Olympic and Paralympic games. World Leis. J. 64, 361–382. doi: 10.1080/16078055.2022.2052951

[ref70] Thompson E. R. (2007). Development and validation of an internationally reliable short-form of the positive and negative affect schedule (PANAS). J. Cross-Cult. Psychol. 38, 227–242. doi: 10.1177/0022022106297301

[ref71] Tricco A. C. Lillie E. Zarin W. O'Brien K. K. Colquhoun H. Levac D. . (2018). PRISMA extension for scoping reviews (PRISMA-ScR): checklist and explanation. Ann. Intern. Med. 169, 467–473. doi: 10.7326/M18-0850 30178033

[ref72] Villalonga-Olives E. Kawachi I. (2017). The dark side of social capital: a systematic review of the negative health effects of social capital. Soc. Sci. Med. *194*, 105–127. doi: 10.1016/j.socscimed.2017.10.020 29100136

[ref73] Watson D. Clark L. A. Tellegen A. (1988). Development and validation of brief measures of positive and negative affect: the PANAS scales. J. Pers. Soc. Psychol. 54, 1063–1070. doi: 10.1037/0022-3514.54.6.1063 3397865

[ref74] Williams D. (2006). On and off the’Net: scales for social capital in an online era. J. Comput.-Mediat. Commun. 11, 593–628. doi: 10.1111/j.1083-6101.2006.00029.x

[ref75] Winkelmann R. (2009). Unemployment, social capital, and subjective well-being. J. Happiness Stud. 10, 421–430. doi: 10.1007/s10902-008-9097-2

[ref76] Xing Z. J. (2003). Developing the brief subjective well-being scale for Chinese citizen. Chin. J. Behav. Med. Sci. 12, 703–705.

[ref77] Xu L. Guo M. Nicholas S. Sun L. Yang F. Wang J. (2020). Disease causing poverty: adapting the Onyx and Bullen social capital measurement tool for China. BMC Public Health 20, 1–10. doi: 10.1186/s12889-020-8163-5 31937283 PMC6961236

[ref78] Yun H. J. (1993). A perception of everyday stress and social network support in adolescence (Master’s thesis). Seoul, South Korea: Seoul National University of graduation.

[ref79] Zhang X. Wang D. Li F. (2022). Physical exercise, social capital, Hope, and subjective well-being in China: a parallel mediation analysis. Int. J. Environ. Res. Public Health *20*:303. doi: 10.3390/ijerph20010303 36612625 PMC9819114

[ref80] Zhou R. Kaplanidou K. Wegner C. (2021). Social capital from sport event participation: scale development and validation. Leis. Stud. 40, 612–627. doi: 10.1080/02614367.2021.1916832

[ref81] Zimet G. D. Dahlem N. W. Zimet S. G. Farley G. K. (1988). The multidimensional scale of perceived social support. J. Pers. Assess. 52, 30–41. doi: 10.1207/s15327752jpa5201_2 2280326

